# The Effect of Bone Mechanical Stress Caused by Electrical Stimulation-Induced Muscle Contraction on Osteocalcin Secretion

**DOI:** 10.3390/biology13110882

**Published:** 2024-10-30

**Authors:** Yi-Chen Chen, Ryoya Oga, Takahiro Furumi, Koki Nakagawa, Yoshihiro Nita, Hiroyuki Tamaki

**Affiliations:** Department of Sports and Life Science, National Institute of Fitness and Sports in Kanoya, Kanoya 891-2393, Japan; m247001@sky.nifs-k.ac.jp (R.O.); m246013@sky.nifs-k.ac.jp (T.F.); m227005@sky.nifs-k.ac.jp (K.N.); m236007@sky.nifs-k.ac.jp (Y.N.); tamaki@nifs-k.ac.jp (H.T.)

**Keywords:** electrical stimulation frequency, muscle contraction, bone strain, undercarboxylated osteocalcin

## Abstract

Electrical stimulation-induced muscle contraction (ESMC) has demonstrated various physiological benefits, but its effects on the secretion of undercarboxylated osteocalcin (ucOC), a bone-derived cytokine, remain unclear. This study explored the relationship between ESMC, bone strain, and ucOC secretion through two experiments. In the first, young male Fischer 344 rats were divided into three groups: low-frequency ES (LF, 10 Hz), high-frequency ES (HF, 100 Hz), and control (CON). Acute 30-min transcutaneous ES was applied, and both bone strain and ucOC levels were measured. In the second experiment, rats underwent LF or HF long-term ES (two sessions per week for 4 weeks), with ucOC and insulin levels monitored. Results revealed a significant peak in ucOC at 6 h post-acute LF-ESMC. Despite HF-ESMC generating greater bone strain, LF-ESMC, with smaller but repetitive bone strain, proved more effective in stimulating ucOC secretion. In the long-term study, both ESMC groups exhibited early increases in ucOC, with a positive correlation to insulin levels. In conclusion, bone strain induced by ES-mediated muscle contraction promotes ucOC secretion, with both the magnitude and frequency of strain playing critical roles.

## Simple Summary

Electrical stimulation-induced muscle contraction (ESMC) is a well-known rehabilitation method used to prevent muscle loss. During exercise, muscle contractions create strain on bones, which can lead to the release of undercarboxylated osteocalcin, a hormone produced by bones that is linked to improved insulin sensitivity and other health benefits. However, it is not clear whether the mechanical stress on bones caused by different frequencies of ESMC can effectively promote this hormone’s release. In this study, we explored how bone strain from both low- and high-frequency ESMC impacts osteocalcin levels. Our results show that ESMC-induced bone strain does promote osteocalcin release, and that this increase is closely related to higher insulin levels. Interestingly, the frequency of stimulation plays a key role, with low-frequency ESMC being particularly effective. These findings suggest new possibilities for rehabilitation and training programs, as low-frequency electrical muscle stimulation is less painful while still producing frequent bone strain, making it a promising approach for improving health outcomes.

## 1. Introduction

Electrical stimulation (ES), a technique for generating passive muscle contraction through external electrical stimuli, has been used in rehabilitation to counteract muscle atrophy by increasing muscle mass, enhancing muscle force, and promoting myofiber regeneration, particularly in individuals with spinal cord injuries (SCI) [1,2]. Beyond its muscular benefits, ES-induced muscle contraction (ESMC) also generates bone mechanical stress and strain, thereby reducing bone loss in disused limbs [3] and increasing sublesional bone mineral density (BMD) near areas of maximum mechanical loading in SCI patients [4].

Evidence from both animal and human studies has demonstrated that exercise enhances secretion of osteocalcin (OC), a bone-derived hormone [5,6], which in turn affects other organs. OC is secreted by osteoblasts and exists in two forms: carboxylated osteocalcin (cOC) and undercarboxylated osteocalcin (ucOC). Both forms are released after transcription and splicing, but cOC, containing three γ-carboxyglutamic acid residues, has a higher affinity for bone matrix, whereas ucOC, with a lower affinity, is more readily released into the bloodstream [7]. When bone resorption occurs, osteoclast activity creates an acidic environment that removes carboxylated residues, converting cOC to ucOC, which is then released into circulation [8]. While both forms of OC are present in the bloodstream, only ucOC exerts hormonal effects [9] and plays important roles in insulin secretion and sensitivity, glucose metabolism in myofibers, fat metabolism, muscle protein synthesis, brain development, cognitive function, testosterone secretion, and male fertility [5,9,10,11,12]. During exercise, muscle contractions produce strain on the skeleton [13], and this mechanical stress can stimulate osteoblasts, promoting the mRNA expression of OC [14,15]. However, it remains unclear whether the bone mechanical stress produced by ESMC effectively promotes OC secretion.

Theoretically, higher intensity or frequency of ES should induce stronger muscle contraction force due to increased muscle fiber recruitment and/or the accumulation of twitch contractions, leading to tetanic contraction and consequently larger mechanical stress on the bone. Previous research indicates that under constant intensity, higher ESMC frequencies result in greater bone strain, with a plateau occurring at 100–150 Hz [16]. Moreover, in suspension animal models, high-frequency ESMC at 100 Hz has shown potential for mitigating muscle atrophy [17]. However, other studies suggest a nonlinear relationship between ESMC frequencies (1 to 100 Hz) and bone strain, with the largest strain occurring at 10 Hz [18]. Lower-frequency ESMC at 10 Hz (incomplete tetanus) has also been shown to reverse muscle mass loss, myofiber cross sectional area (FCSA), and bone loss in the disused limbs of both young and elder rats [3,19].

While it has been established that the magnitude of bone strain generated by ESMC is related to the ES pattern, this relationship is not yet fully defined. Both low- and high-frequency ESMC interventions appear to improve muscle atrophy and apply mechanical stress to bones, but it is still unknown whether higher or lower frequency ESMC is more effective at promoting ucOC secretion. Moreover, the impact of long-term ESMC on ucOC secretion remains unclear. Understanding the effects of bone strain caused by acute ESMC on ucOC, as well as developing a long-term ESMC intervention protocol, could provide substantial societal benefits.

Therefore, this study aimed to (1) investigate the magnitude of bone strain elicited by both low- and high-frequency ESMC and the temporal effects on ucOC concentration and (2) design a long-term ES intervention protocol based on these temporal effects, to assess its long-term impacts on muscle and ucOC. Whether used as a rehabilitation tool for individuals with bedridden conditions, osteoporosis, sarcopenia, those who are unable to exercise, as a method of health promotion for the general population, or as a supportive training strategy for athletes, exploring the effects of ESMC holds extensive potential for future applications.

## 2. Materials and Methods

### 2.1. Animals and Experimental Protocol

Male Fischer 344 rats (CLEA, Tokyo, Japan) were housed in cages (1–2 rats per cage) under a 12-h light-dark cycle, with constant temperature (23 ± 2 °C) and humidity (50 ± 5%). The rats were fed CE-2 rodent chow (CLEA) and water *ad libitum*.

In the first study, in vivo bone strain values induced by ESMC were measured in the tibia of rats (n = 3) under inhalational anesthesia (1.5–2.5% isoflurane, 2 L/min flow rate). Subsequently, 22 rats, aged 10 weeks, were randomly assigned to three groups: (1) control (CON, n = 6), (2) low-frequency ESMC at 10 Hz (LF, n = 8), and (3) high-frequency ESMC at 100 Hz (HF, n = 8). The sample size was calculated based on previous serum ucOC data using G*Power software 3.1 (Heinrich-Heine-Universität Düsseldorf, Düsseldorf, Germany), with a two-tailed significance level of 5% and a power of 80%. The ESMC groups underwent an acute high-intensity ES intervention at either 10 Hz or 100 Hz frequency under isoflurane anesthesia. The CON group underwent inhalation anesthesia under the same conditions without ES. To investigate the temporal changes in circulating ucOC concentrations following a single ES intervention, blood samples were collected at multiple time points post-intervention.

Based on the findings from the first study, a second study was conducted to examine the long-term effects of ES. Another 22 rats (aged 12 ± 2 weeks) were divided into the same groups as in the first study. The ESMC groups underwent ES interventions twice per week for 4 weeks (eight sessions in total) under the same ES conditions as the first study. Blood samples were collected before the first session and 6 h after the first, fourth, and eighth sessions for ucOC and insulin measurement. Isometric contraction force was measured 24 h after the last ES session, and the rats were sacrificed to obtain muscle samples. All data were analyzed in a blinded fashion, with the identification codes revealed only after data analysis was completed. None of the animals experienced a 20% reduction in body weight. All procedures were approved by the Institutional Animal Care and Use Committee of the National Institute of Fitness and Sports in Kanoya (approval number: R05-02).

### 2.2. Direct ES Procedures

The stimulation protocol was delivered as previously described [19]. Transcutaneous ES was performed on the left tibialis anterior (TA) muscle under isoflurane inhalational anesthesia (1.5–2.5% isoflurane, 2 L/min flow rate). Bipolar silver surface electrodes (4 mm diameter) were adhered to the shaved skin surface of the left TA muscle. An acute intervention was performed using an electro stimulator and isolators (SEN-8203MG, SS-104J, Nihon Kohden, Tokyo, Japan) to apply direct ES at 20 ± 2 V intensity, with 10 Hz or 100 Hz frequency, 250 μs pulse width, and 2 s of stimulation followed by 6 s of rest, for a total 30-min session.

### 2.3. Tibia Bone Strain Measurement

In vivo tibia bone strain during ES-induced muscle contraction was measured following the protocol of a previous study [3]. Under isoflurane inhalational anesthesia, after removing soft tissue at the adhesion site, strain gages (KFG-3-120, Kyowa, Tokyo, Japan) were longitudinally attached to the middle surface of the tibia using cyanoacrylate instantaneous adhesive (CC-33A, Kyowa, Tokyo, Japan), pressed for one minute to ensure complete adhesion. Strain signals were amplified (DPM-951A, Kyowa, Tokyo, Japan) and sampled at 2 kHz via an A/D converter (PL3508 PowerLab 8/35, ADInstruments, Nagoya, Japan).

### 2.4. Blood Samples Collection and Measurement

Blood samples were collected from the caudal vein after fasting for more than 9 h. After clotting at room temperature for 30 min, the blood was centrifuged at 3000× *g* for 30 min at 4 °C. The supernatant was collected and centrifuged again for 10 min under the same conditions, and the serum was separated and stored at −80 °C until analysis. A second centrifugation was conducted as needed. Enzyme-linked immunosorbent assays (ELISA) were used to measure circulating osteocalcin and insulin concentrations. Osteocalcin levels were measured using the rat Glu-osteocalcin high-sensitive EIA kit (MK146, TaKaRa Bio Inc., Shiga, Japan), and insulin levels were measured using the Morinaga ultra-sensitive rat insulin ELISA kit (M1103, Morinaga Institute of Biological Science, Inc., Yokohama, Japan).

### 2.5. Muscle Contraction Force Measurement and Muscle Sample Harvest

After the long-term ES intervention, isometric contraction force of the TA muscle was measured as described previously [20,21]. Rats were anesthetized with isoflurane, and their lower limbs were stabilized on the working platforms using restraining bars and pins at the knee and ankle joints. The distal tendon of the TA muscle was attached along the natural pull of the muscle to an isometric transducer (TB-654T, Nihon Kohden, Japan), which was secured to a three-dimensional drive precision stage with a 3-0 silk suture. Bipolar silver electrodes were attached to the shaved skin surface of the TA muscle, which was connected to the electro-stimulator and isolators (SEN-8203MG, SS-104JMG, Nihon Kohden, Tokyo, Japan) to produce ES. The muscle tension signals were sampled at 2 kHz through an A/D converter (PL3508 PowerLab 8/35, ADInstruments, Nagoya, Japan). Rats were sacrificed under anesthesia, and the TA and soleus (SOL) muscle samples were harvested and weighed. The middle section of the TA muscle was mounted vertically on cork with OCT compound, frozen in isopentane cooled in liquid nitrogen, and stored at −80 °C.

### 2.6. Immunohistochemical Analysis

The TA muscle samples were sectioned (10 µm) using a cryostat (CM3050S, Leica, Nussloch, Germany) at −20 °C and mounted on silanized slides. After air drying at room temperature, the tissue was fixed with 4% paraformaldehyde (PFA) for 15 min and washed twice with 0.1 M phosphate-buffered saline (PBS). The samples were incubated in 100% methanol for 10 min at −20 °C, then washed twice with PBS. Next, the sections were blocked with 10% normal goat serum (NGS) and 1% Triton X-100 in PBS for 1 h at room temperature, before they were removed and incubated in mouse anti-dystrophin antibody (1:250 dilution, D8168, Merck, Germany) with 0.3% Triton X-100 and 2.5% NGS in PBS for 16–20 h at 4 °C. After washing, the sections were washed several times with PBS and incubated in the appropriate secondary antibodies (Alexa Fluor 488 goat anti-mouse IgG antibody, 1:500 dilution, ab150117, Abcam, Japan) with 0.1% Triton X-100 and 2.5 % NGS in PBS for 1 h at room temperature. Following PBS washes, the sections were mounted using Vectashield. A fluorescence microscope (BZ-X710, Keyence, Osaka, Japan) was used to obtain images of the TA muscle sections. Each muscle section was divided into five equal parts, and at least 30 muscle fiber areas were measured in each part for a total of at least 150 muscle fibers being assessed for myofiber cross-sectional area (FCSA) using Image-Pro Premier software (Media Cybernetics, Rockville, MD, USA).

### 2.7. Statistical Analysis

Statistical analyses were performed using IBM SPSS Statistics version 25 (IBM, Chicago, IL, USA). All data are presented as the mean ± standard deviation (SD). Normality of the data distribution was confirmed using the Shapiro–Wilk test. Parametric or non-parametric statistical methods were applied depending on the normality of the data distribution. Differences between groups were analyzed using one-way analysis of variance (ANOVA) followed by Tukey’s post-hoc test or Kruskal-Wallis test followed by Mann−Whitney U test. Two-way ANOVA was used to assess the effects of intervention and time, followed by Tukey’s post-hoc test. Pearson correlation was used to assess the correlation between ucOC and insulin concentration. *p*-values < 0.05 were considered statistically significant. Hedges’ *g* effect size and the 95% confidence intervals (95% CIs) for intergroup ucOC levels in the first study were calculated.

## 3. Results

### 3.1. Bone Strain Induced by ESMC and ucOC Levels after a Single ESMC Session

**Figure 1:**
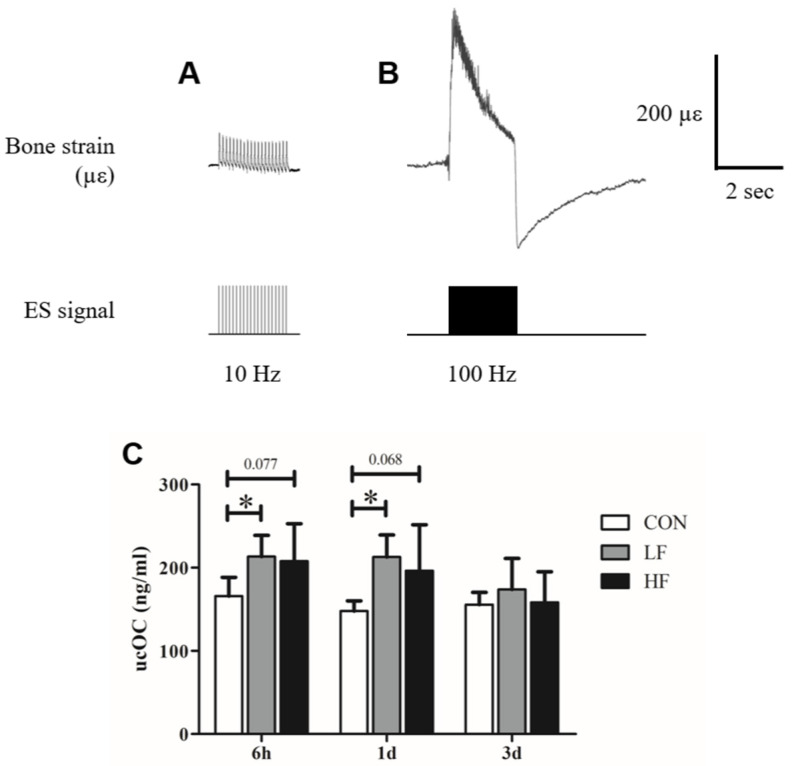
Bone strain under (**A**) 10 Hz, (**B**) 100 Hz electrical stimulation-induced muscle contraction (ESMC), and (**C**) the temporal changes of ucOC after a single bout of ESMC intervention. * *p* < 0.05. (CON, control [n = 6]; LF, low-frequency electrical stimulation [n = 8]; HF, high-frequency electrical stimulation [n = 8]). Values are presented as the mean ± SD.

The results of the first study are presented in Figure 1. Under 10 Hz ESMC, smaller but more repetitive bone strains were observed, with a maximum strain of 43 ± 5 µε (Figure 1A). In contrast, 100 Hz ESMC induced bone strains that occurred almost synchronously with stimulation, rapidly rising to 204 ± 98 µε before slightly declining and then dropping sharply to a negative value at the end of the 2-s stimulation period, eventually returning to baseline (Figure 1B). The mean strain rates for 10 Hz and 100 Hz ESMC were 3655 ± 436 µε/s and 3075 ± 682 µε/s, respectively. The ucOC levels significantly increased 6 h after a single ES intervention in the LF group compared with the CON group (Hedges’ *g* = 1.95, 95% CIs = −93.26 to −1.50, *p* = 0.042), while an increasing trend was observed in the HF group (Hedges’ *g* = 1.12, 95% CIs = −87.76 to 4.00, *p* = 0.077). The significant increase in the LF group (Hedges’ *g* = 3.02, 95% CIs = −116.80 to −13.48, *p* = 0.012) and the rising trend in the HF group (Hedges’ *g* = 1.13, 95% CIs = −100.13 to 3.19, *p* = 0.068) persisted until 1 day post-intervention before gradually decreasing. By 3 days post-intervention, no significant difference was observed between the ES and CON groups (Figure 1C).

### 3.2. UcOC and Muscle Analysis of the Long-Term ESMC Intervention

Following the 4-week ES intervention, there were no significant differences in body weight, TA and SOL muscle weight, relative muscle weight, or TA muscle contraction force between the groups (Table 1).

**Figure 2:**
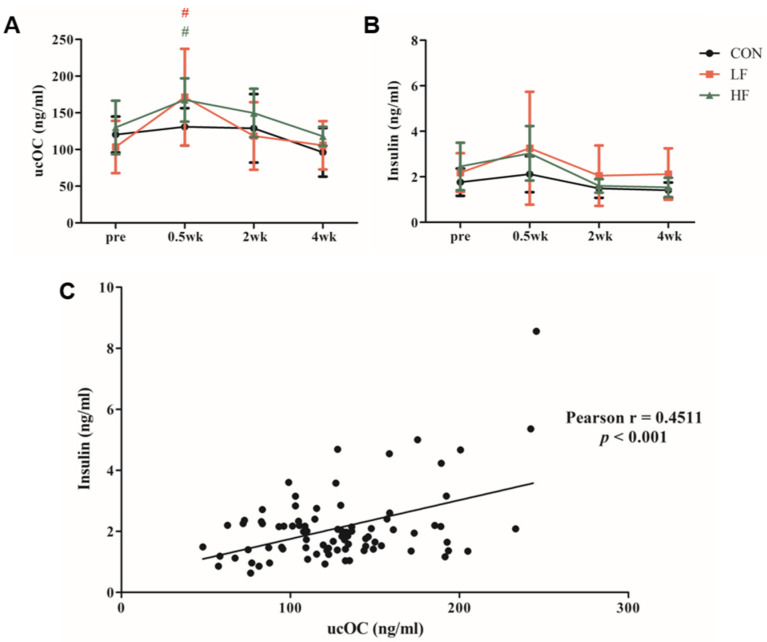
The changes in (**A**) ucOC, (**B**) insulin levels during the 4-week ESMC intervention, and (**C**) the correlation between ucOC and insulin levels at all time points. # *p* < 0.05 vs. pre. (CON, control [n = 6]; LF, low-frequency electrical stimulation [n = 8]; HF, high-frequency electrical stimulation [n = 7]). Values are presented as the mean ± SD.

In the long-term ES study, no significant differences were found in baseline ucOC or insulin levels between groups. After the first (0.5 week) ES session, serum ucOC levels were significantly elevated from baseline in both the LF (*p* = 0.012) and HF groups (*p* = 0.007) (Figure 2A). However, no significant differences were observed after the 2nd and 4th weeks of ES interventions. Insulin levels showed no significant differences between or within groups at any time point (Figure 2B). Nonetheless, a significant positive correlation was found between ucOC and insulin levels across all time points (r = 0.4511, *p* < 0.001) (Figure 2C).

**Figure 3:**
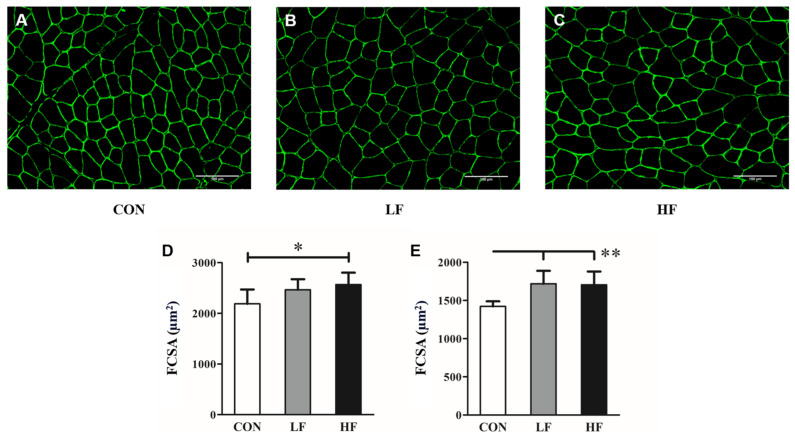
Immunohistochemical staining photomicrographs of tibial anterior (TA) muscle for dystrophin in (**A**) CON, (**B**) LF, and (**C**) HF groups, and quantification of mean myofiber cross-sectional area (FCSA) for (**D**) all sections and (**E**) deep sections. The scale bar represents 100 μm. * *p* < 0.05; ** *p* < 0.01. (CON, control [n = 6]; LF, low-frequency electrical stimulation [n = 8]; HF, high-frequency electrical stimulation [n = 8]). Values are presented as the mean ± SD.

Immunohistochemical staining of cross-sectional images (Figure 3A–C) revealed that the mean myofiber cross-sectional area (FCSA) of the TA muscle was significantly larger in the HF group compared to the CON group (*p* = 0.023) (Figure 3D). When focusing on the deep part of the TA muscle (closer to the bone), both the LF (*p* = 0.005) and HF (*p* = 0.007) groups displayed significantly larger FCSA compared to the CON group (Figure 3E), indicating that transcutaneous ES stimulated deeper muscle layers, leading to complete muscle contraction.

## 4. Discussion

**Figure 4:**
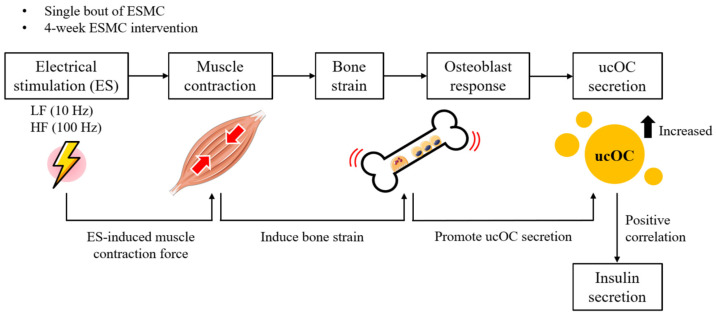
The schematic summary of the methodology and research findings. (LF, low-frequency electrical stimulation; HF, high-frequency electrical stimulation; ESMC, electrical stimulation-induced muscle contraction; ucOC, undercarboxylated osteocalcin).

This study provides novel insights into the effects of single and long-term low- and high-frequency transcutaneous ESMC on ucOC, insulin levels, and muscle changes. Our findings demonstrate that bone strain can be induced in synchrony with electrical stimulation, with LF-ESMC eliciting repetitive but smaller bone strain, and HF-ESMC generating single but larger bone strain patterns. In terms of promoting ucOC secretion, although both bone strain patterns induced ucOC secretion temporally, LF-ESMC appeared to be more effective. In the long-term study, significant increases in ucOC were only observed in the early phase of both LF and HF-ESMC interventions. While insulin levels did not change significantly during the long-term ES intervention, a positive correlation between ucOC and insulin was observed at all time points. Additionally, the increased FCSA in the deeper part of the muscle in both LF and HF groups suggests that the electrical stimulus effectively reached deeper muscle layers, leading to complete muscle contraction. The summary of methodology and research findings is shown in Figure 4.

The finding that ES-induced muscle contraction leads to bone strain is consistent with previous studies [3,16] showing that higher ES frequencies can produce greater bone strains. However, the magnitude of bone strain at 100 Hz in our study was smaller than that observed in previous research using mice under the same conditions [16]. Several factors could account for this difference, including species variation and the specific muscle groups contracted. Mice have lower cortical bone thickness and elastic modulus compared to rats [22,23], making their bones more susceptible to strains. Furthermore, previous studies focused on ES applied to the tibia nerve, primarily contracting the soleus and gastrocnemius muscle, whereas the present study stimulated the TA muscle. The contraction of larger, multi-muscle groups may cause a greater bone strain, which could explain the smaller bone strain observed in our study.

Bone strain responses are known to be linearly related to peak dynamic loads within the anabolic strain region [24]. A threshold of approximately 1050 µε is required to activate bone cells and stimulate bone formation [25]. Once the threshold is exceeded, the rate of bone formation increases linearly with enhanced bone loading and strain. Turner and colleagues [25] noted that different intervention conditions may yield different results. In their study, a four-point loading apparatus was employed to apply bending loads to the tibia, which differs from our approach. Furthermore, their subjects were 9-month-old female rats, highlighting how factors such as gender and age can influence bone characteristics. Sex hormones play an important role in bone growth, with androgens mediating periosteal growth. Higher androgen levels in males contribute to greater cortical thickness and diameter, resulting in a greater resistance to bending [26]. Consequently, female bones may be more susceptible to evoked bone strain. Furthermore, the threshold for strain-induced responses may be elevated in conditions where bone strain is more easily achieved. Aging also impacts bone mechanical responsiveness. Osteocytes, the predominant mechanosensory cells in bone, detect mechanical strains and relay signals to osteoblasts for appropriate responses [27]. However, aging leads to osteocyte apoptosis and shrinkage, diminishing their sensitivity to mechanical stress [6,28,29].

Although few studies have evaluated bone strain patterns under different exercise modes, previous research has demonstrated that muscle contraction forces create mechanical stimulation on bone [13,30]. Notably, over 70% of the force transmitted to the femur is generated by muscle contraction forces, while less than 30% derives from the ground reaction force during normal gait [31]. Therefore, bone strain patterns can be inferred from the patterns of exercise-induced muscle contraction. During resistance exercise, short-term, high-intensity muscle contractions may lead to larger but less repetitive bone strains. In contrast, aerobic exercise generates low-intensity, long-duration contractions that are highly repetitive, potentially inducing small but frequent bone strain and enhancing ucOC secretion. This aligns with our research findings, suggesting that while the magnitude of bone strains is important for stimulating ucOC secretion, the number of repetitions is also a crucial factor.

In the present study, bone strain was induced via ESMC, with the bone strain pattern directly influenced by the muscle contraction frequency. Summation of muscle contractions typically began around 20–30 Hz ES, leading to tetanic contractions at approximately 100 Hz, which resulted in fewer bone strain repetitions. Both bone strain pattern and strain rate are crucial for enhancing ucOC secretion, as higher strain rates are generally more osteogenic [32]. Warden and colleagues [33] demonstrated that applying axial loading to the ulna at peak loads of 1.5 and 2.0 N across frequencies of 1–30 Hz increased bone formation, particularly at the higher load (2.0 N) across all frequencies. Interestingly, with the lower load (1.5 N), significant increases in bone formation were observed at 10 Hz and 20 Hz, indicating that lower-frequency stimulation with smaller strain magnitudes can also effectively promote bone formation. However, the optimal frequency for maximizing bone formation remains uncertain, warranting further investigation. Considering that ESMC-induced tetanic contractions limit strain repetitions, direct bone stimulation may achieve higher strain rates and repetitions, though with minimal influence on muscle tissue. ESMC, on the other hand, provides simultaneous mechanical stimulation to bone and induces muscle contractions, influencing both tissues. Future studies should account for this dual impact when developing intervention protocols.

Several studies have indicated that ucOC improves insulin secretion and sensitivity [7,9,34]. For instance, Ferron and colleagues [34] found that ucOC’s effect on insulin expression in pancreatic β-cells is concentration-dependent, with an optimal dose of 0.3 ng/mL. The change in ucOC observed in our study, standardized by body weight, aligns with this optimal dose, suggesting that the increase in ucOC due to ES intervention should be sufficient to promote insulin secretion. However, the expected results were not achieved; despite a positive correlation between ucOC and insulin concentration, no significant changes in insulin levels were noted during the intervention. This discrepancy may stem from the complex interactions between ucOC and insulin, both of which are influenced by other hormones such as leptin and growth hormone [35,36]. Future studies should evaluate these additional hormones to better understand the relationship between ES, ucOC, and insulin.

In the long-term ES intervention, a significant increase in ucOC was observed only in the early phase, with no significant changes thereafter. This may be influenced by several factors, including the balance between osteoblast and osteoclast, physiological changes in bone, and the temporal dynamics of ucOC secretion. Bone remodeling is a complex process influenced not only by mechanical stress but also by factors like exercise-induced inflammatory response and irisin secreted by skeletal muscle during physical activity [37]. In the present study, no significant changes in muscle weight and muscle force after the 4-week intervention compared to the CON group may suggest that the muscle contraction forces applied to the bone remained consistent throughout the intervention period. Prolonged stimulation may result in decreased osteogenic responsiveness [38]. The absence of a significant increase in ucOC during the later stages of the intervention may indicate reduced osteoblast sensitivity, referring to the diminished bone formation response following prolonged loading [25]. Moreover, Turner and colleagues [25] also noted that while bone strain above a certain threshold promotes bone formation, increased bone volume may result in reduced strain production, indicating that bone might become less responsive to the same ES intervention over time. In summary, changes in bone remodeling and mechanical load responsiveness may affect temporal variations in ucOC levels, potentially shifting the peak secretion time with prolonged interventions. Future studies tracking alterations in bone tissue and temporal changes in ucOC following long-term ES intervention could provide valuable insights.

Although the primary objective of this study was to investigate the effects of ESMC-induced bone strain on ucOC secretion, post-intervention muscle parameters were also evaluated to confirm that the protocol did not result in muscle atrophy or strength loss. The findings showed an increase in FCSA without significant changes in muscle weight or force. While the underlying physiological mechanisms are not fully understood, a reduction in intermuscular adipose tissue (IMAT) associated with exercise [39] may explain the stable muscle weight despite an increase in FCSA. Furthermore, a longer intervention period could potentially impact strength increases [40]. Overall, these results indicate that the intervention protocol did not negatively impact muscle weight or force.

This study has several limitations. Firstly, we did not assess other hormones that could influence ucOC and insulin levels or bone remodeling, such as leptin, growth hormone, and irisin, which may have direct or indirect effects on ucOC secretion and its downstream impacts. Evaluating these hormones could provide a more comprehensive understanding of the physiological mechanisms involved. Secondly, no bone structural analysis was conducted following the long-term ES intervention, which may play a role in mediating the effects of ESMC on ucOC secretion. Further studies are needed to explore these mechanisms in more detail.

## 5. Conclusions

In conclusion, this study demonstrates that bone strain induced by ES-induced muscle contraction effectively stimulates ucOC secretion in young rats. Notably, the 10 Hz ESMC intervention proved more effective than that at 100 Hz, highlighting that beyond the magnitude of bone strain, the frequency of stimulation is a critical factor in promoting ucOC secretion. Furthermore, the 10 Hz ESMC is milder and less painful than the 100 Hz option, making it a promising approach for future applications in rehabilitation and training. While these findings offer valuable insights with potential societal implications, further research is necessary to elucidate the underlying mechanisms and long-term effects.

## Figures and Tables

**Table 1:** Body weight (BW) and muscle weight (MW) after the 4-week ESMC intervention.

		CON	LF	HF	*p*
Body weight	(g)	258.50 ± 15.61	259.88 ± 15.08	257.50 ± 16.36	0.961
TA muscle weight	(mg)	485.50 ± 46.33	495.00 ± 54.97	495.00 ± 38.61	0.927
Relative muscle weight	(MW/BW)	1.88 ± 0.09	1.90 ± 0.11	1.92 ± 0.08	0.700
Soleus muscle weight	(mg)	101.67 ± 10.87	104.00 ± 9.29	105.25 ± 8.84	0.814
Relative muscle weight	(MW/BW)	0.39 ± 0.03	0.40 ± 0.02	0.41 ± 0.02	0.466
TA muscle force	(N)	7.68 ± 0.29	7.64 ± 1.03	7.34 ± 0.8	0.754

Values are presented as mean ± SD.

## Data Availability

The original contributions presented in the study are included in the article, further inquiries can be directed to the corresponding author/s.
